# Ranking’s Half-Yearly Abstract

**Published:** 1848-04

**Authors:** 


### RANKING'S HALF-YEARLY ABSTRACT.

We have received the sixth number of this valuable Abstract of the Medical Sciences, and are able to state that the work maintains the high character which it has attained among the periodical publications of the day. We do not see how physicians who are not readers of the Half-Yearly Abstract, or of some medical journal, can pretend to know whether our profession is making any progress. With such a multitude of journals before them, practitioners in America are inexcusable who neglect to avail themselves of the rich source of instruction afforded by these publications.

